# Genotyping-by-Sequencing Based Molecular Genetic Diversity of Pakistani Bread Wheat (*Triticum aestivum* L.) Accessions

**DOI:** 10.3389/fgene.2022.772517

**Published:** 2022-04-06

**Authors:** Shabbir Hussain, Madiha Habib, Zaheer Ahmed, Bushra Sadia, Amy Bernardo, Paul St. Amand, Guihua Bai, Nida Ghori, Azeem I. Khan, Faisal S. Awan, Rizwana Maqbool

**Affiliations:** ^1^ Center of Agricultural Biochemistry and Biotechnology, University of Agriculture, Faisalabad, Pakistan; ^2^ Department of Plant Breeding and Genetics, University of Agriculture, Faisalabad, Pakistan; ^3^ USDA, Hard Winter Wheat Genetics Research Unit, Manhattan, KS, United States

**Keywords:** genotyping-by-sequencing, genetic diversity, population structure, *Triticum aestivum* L., single nucleotide polymorphisms, polymorphic information content, Pakistan

## Abstract

Spring wheat (*Triticum aestivum* L.) is one of the most imperative staple food crops, with an annual production of 765 million tons globally to feed ∼40% world population. Genetic diversity in available germplasm is crucial for sustainable wheat improvement to ensure global food security. A diversity panel of 184 Pakistani wheat accessions was genotyped using 123,596 high-quality single nucleotide polymorphism (SNP) markers generated by genotyping-by-sequencing with 42% of the SNPs mapped on B, 36% on A, and 22% on D sub-genomes of wheat. Chromosome 2B contains the most SNPs (9,126), whereas 4D has the least (2,660) markers. The mean polymorphic information content, genetic diversity, and major allele frequency of the population were 0.157, 0.1844, and 0.87, respectively. Analysis of molecular variance revealed a higher genetic diversity (80%) within the sub-population than among the sub-populations (20%). The genome-wide linkage disequilibrium was 0.34 Mbp for the whole wheat genome. Among the three subgenomes, A has the highest LD decay value (0.29 Mbp), followed by B (0.2 Mbp) and D (0.07 Mbp) genomes, respectively. The results of population structure, principal coordinate analysis, phylogenetic tree, and kinship analysis also divided the whole population into three clusters comprising 31, 33, and 120 accessions in group 1, group 2, and group 3, respectively. All groups were dominated by the local wheat accessions. Estimation of genetic diversity will be a baseline for the selection of breeding parents for mutations and the genome-wide association and marker-assisted selection studies.

## Introduction

Wheat (*Triticum aestivum* L.) is amongst the most prominent cereal crops in the world. Wheat is cultivated on more than 224 million hectares (Mha) and provides 20% (Bhatta et al., 2017) of dietary nutrients for 40% world population (Ray et al., 2013; Arzani and Ashraf, 2017; Food and Agriculture Organization of the United Nations, 2019). Moreover, 68% of global wheat is directly utilized for human food while the remaining 32% for livestock consumption and other miscellaneous uses such as biofuel (Mohanty and Swain, 2019). The world population is increasing at the rate of 1.5% per annum and is expected to reach 9.7 billion by 2050 (Lobell et al., 2011). However, a 0.9% annual increase in bread wheat yield currently is not sufficient to ensure global food security (Ray et al., 2013). Pakistan is the fifth densely populated country in the world. Wheat was cultivated on an area of 8,825 thousand ha of land, which produced 24,946 MT of grain in 2020 (Pakistan Economic Survey 2019-20).

Wheat genetic gains can be improved by optimizing crop husbandry (Sener et al., 2009) and harnessing genetic diversity in native germplasm resources (Nielsen et al., 2014; Govindaraj et al., 2015). Dissection of genetic diversity is a prerequisite for plant breeding experiments, such as domestication, inheritance, conservation, and evaluation of wheat germplasm (Peterson et al., 2014). Narrow genetic bases, limited genetic diversity, and continuous reduction in arable farmland, as well as various climate-associated anomalies in the form of biotic and abiotic stresses, pose a continuous threat to world food security in developing countries (Fischer et al., 2014). Moreover, wheat has a long evolutionary history from the days of its early domestication from Einkorn (one of the primitive wheat ancestors) to modern bread wheat and assimilates plenty of genetic variation during this long period of evolution. Farmers’ selection, uniform varietal seed production, continuous selfing, use of modern breeding techniques, domestication, and stringent selection pressure lead to prompt genetic erosion and squat gene pool, which causes continuous losses of favorable alleles in currently used wheat germplasm (Haudry et al., 2007; Sofalian et al., 2008).

Strategic trait-based wheat breeding is the most viable and sustainable solution for crop improvement. The success of a wheat breeding program can be directly associated with the availability of valuable genetic diversity in the program (Rufo et al., 2019; Voss-Fels et al., 2019). Genetic resources such as wild relatives, gene bank accessions, landraces, advanced breeding lines, and induced and natural mutants are considered indispensable genetic resources for maintaining genetic diversity and crop improvement (Ogbonnaya et al., 2013; Das et al., 2016). Natural variation selection, wide crossing, new gene introduction, genetic hybridization, induced mutagenesis, and horizontal and vertical gene transfer can contribute to enriching the genetic diversity of modern wheat varieties to meet the challenges of climate change and global food security (Arya et al., 2013).

DNA markers are considered indispensable tools for the genetic characterization of plants. Various types of DNA-based markers, including randomly amplified polymorphic DNA (RAPD), amplified fragment length polymorphisms (AFLP), restriction fragment length polymorphisms (RFLP), and simple sequence repeats (SSRs) (Röder et al., 1995), were successfully used for genetic characterization in plants. Nowadays, single nucleotide polymorphism (SNP) markers generated through next-generation sequencing (NGS,) such as targeted amplicon sequencing (TAS), Illumina bead chip array, DArT, Genotyping-by-Sequencing (GBS), and kompetitive allele-specific PCR (KASP), are gradually replacing other old marker systems due to high throughput and low cost per data point. GBS is a reduced representation sequencing method that identifies SNP for genotyping and discovers new SNP compared to other array-based genotyping technologies, namely, DArT/Illumina bead chip. An array-based genotyping chip targets and identifies a pre-labeled specific number of SNPs markers. GBS-based sequencing could be effectively used in the breeding of complex genome crops without any prior sequencing information or even in the absence of reference genome in many orphan crops (Getachew et al., 2019). GBS is a technology that can reduce genome complexity by using two restriction enzymes (*Pst*I/*Msp*I) and simultaneously discover and genotype genome-wide variations in complex genome crops, namely, bread wheat (Elshire et al., 2011; Poland et al., 2012a; Poland et al., 2012b). Currently, GBS has been successfully used in many crops to unblock the GD including wheat, barley, rice, maize, cassava, potato, and soybean (Elshire et al., 2011).

SNPs are the most abundant polymorphic markers in both plant and animal genomes (Rimbert et al., 2018). The bi-allelic nature, high level of polymorphisms, ubiquitous presence, uniform distribution across genomes, automated data acquisition, and analysis make SNPs the most suitable marker for genome-wide marker analysis (Verma et al., 2015). Quick advancement in NGS techniques significantly improved sequencing throughput and reduced sequencing cost, making the genome-wide SNP analysis a time- and cost-effective tool for genomic studies (He et al., 2014). Therefore, the NGS-based markers have been widely used for retrieving genetic diversity (GD), harnessing population structure (PS), studying linkage disequilibrium (LD), mapping quantitative trait loci (QTL), conducting genome-wide association studies (GWAS), and genomic selection (GS) in various crops (You et al., 2018).

Although the cost of whole-genome sequencing significantly reduced during the last decade, still it is not feasible to completely sequence all genotypes for routine screening of breeding materials especially for those species with a huge genome such as wheat in most breeding programs. Mourad et al. (2020) genotyped a panel of 103 spring wheat accessions that were collected from five continents and deposited in the USDA gene bank to evaluate the GD, PS, and LD patterns. The panel was genotyped with 36,720 high-quality SNPs, and the whole population was divided into three subpopulations on the basis of analysis of molecular variance (AMOVA), structure analysis, kinship, and principal component analysis. No high LD was observed on a whole bread wheat genome, but at the sub-genomic level, the D genome showed the highest LD decay value compared to the A and B sub-genomes.

Alipour et al. (2017) utilized 16,506 polymorphic GBS-SNPs to dissect the GD of an Iranian wheat diversity panel of 369 wheat genotypes. The B subgenome has the highest number of mapped SNPs compared to the other A and D subgenomes, respectively. The whole population was divided into three subgroups: one for cultivars and two for landraces. Heslot et al. (2013) analyzed 38,412 GBS-SNPs in 365 soft winter wheat to harness GD in advanced breeding lines. Yang et al. (2020) characterized a population of 180 bread wheat genotypes from Asia and Europe to determine the indigenous PS and GD using 24,767 high-quality polymorphic SNPs using the GBS approach and to determine GD of the subjected population. The polymorphic information content (PIC) value of markers ranges from 0.1 to 0.4. Based on cluster and structure analysis, the whole diversity panel was divided into two groups: group 1 comprises European and partial Asian and group 2 consists of the Middle East and partial Asian accessions.

Most Pakistani wheat germplasm has not been characterized using DNA (SNP) markers. Local germplasm is always a key source of resistance against biotic and abiotic anomalies. The primary objective of this study is to investigate the extent and the pattern of GD, PS, LD, PIC, phylogeny, and kinship of the subjected population that will be useful for the selection of breeding parents for various stress breeding strategies through GWAS and association mapping (AM).

## Materials and Methods

### Deoxy Nucleic Acid Isolation and Genotyping-by-Sequencing Library Preparation

A set of 184 Pakistani spring wheat varieties and germplasm accessions were collected from the Wheat Research Institute, Ayub Agricultural Research Institute (AARI), Pakistan, Faisalabad (Supplementary Table S1). The panel was sown into two 96-well plastic trays in the greenhouse at the Kansas State University, Manhattan, KS, United States. Three pieces of 15-day-old seedling leaf tissues (2 cm) were collected in 1.1 ml 96-deep-well plates with 3 mm stainless beads in each well and immediately freeze-dried for 2 days. Leaf tissues were ground by shaking the plates at 30 cycles per second for 3 min in a Mixer Mill (Retsch GmbH, Haan, Germany). Genomic DNA was isolated using the standard cetyltrimethylammonium bromide (CTAB) method with a slight modification (Zhao et al., 2020). DNA quality was checked with 1% agarose gel and quantified in a FLUOstar Omega microplate reader (BMG LABTECH, Germany) using Quant-iT™ PicoGreen dsDNA assay kits (Thermo Fisher Scientific, Waltham, MA, United States). The genomic DNA of all samples was normalized at a concentration of 20 ng/μL for preparing the GBS library.

GBS libraries of 184 spring wheat samples were prepared using the standard protocol (Poland et al., 2012a). In brief, normalized DNA (200 ng) was digested using restriction enzyme *Pst*I-HF and *Msp*I from New England BioLabs, Inc. (Ipswich, MA, United States) and ligated with barcoded adapters using a T4 DNA ligase (New England BioLabs, Inc., Ipswich, MA, United States). Ligated DNA fragments were pooled and purified using QIAquick PCR Purification Kit (QIAGEN GmbH, Hilden, Germany). Primers complementary on both adapters were used for PCR amplification. PCR product was cleaned using QIAquick PCR Purification Kit and size selected using an E-gel (Thermo Fisher Scientific, Waltham, MA, United States) to select 200–300 bp fragments and then quantified using Qubit 2.0 Fluorometer and Qubit dsDNA HS Assay Kits (Life Technologies Inc., Carlsbad, CA, United States).

### Single Nucleotide Polymorphism Calling and Data Imputation

The final library was sequenced in an Ion Proton next-generation sequencer (Thermo Fisher Scientific, Waltham, MA, United States) in the USDA Central Small Grain Genotyping Lab, Kansas State University, Manhattan, KS, United States. SNPs were called using the GBS discovery pipeline v2.0 in Trait Analysis by Association, Evolution, and Linkage (TASSEL) v5.2.63 (Bradbury et al., 2007) by aligning the sequence reads with the International Wheat Genome Sequencing Consortium (IWGSC) reference genome RefSeq V2.0 (IWGSC, 2018). Initially called SNPs were filtered to remove these SNPs with >20% missing data and <0.01% minor allele frequency (MAF) and then imputed for missing data using BEAGLE v5.1, (Browning and Browning, 2009), in TASSEL v5.2.63.

### Genetic Diversity, Polymorphic Information Content, and Analysis of Molecular Variance

TASSEL v5.2.67 was used to calculate the evolutionary relationship among the 184 Pakistani accessions. The dendrogram was constructed using the Neighbor-Joining (NJ) distance method (Saitou and Nei, 1987) in TASSEL. Principal coordinate analysis (PCoA) was implemented using the Euclidean distance method in GenAlEx v6.5 (Peakall and Smouse, 2006). Population GD, PIC, major allele frequency (MF), and percentage heterozygosity (HZ) were determined using POWER MARKER v3.25 (Liu and Muse, 2005). Analysis of molecular variance (AMOVA) (Excoffier et al., 1992) and Shannon’s information index (*I*) were calculated using GenAlEx v6.5 (Peakall and Smouse, 2006).

### Analyses of Population Structure, Linkage Disequilibrium, and Kinship Analysis

Population structure was inferred using an admixture model and Bayesian model-based clustering algorithm in STRUCTURE v2.3.4 (Pritchard et al., 2000). The best fit delta K value for the number of subpopulations was determined using K values from 1 to 10 and burned in 10,000 generations and 100,000 Markov chain Monte Carlo of (MCMC) iterations (Zorić et al., 2012; Chen et al., 2012). The output of STRUCTURE analysis was visualized using a STRUCTURE HARVESTER software (Earl and vonHoldt, 2012). TASSEL v5.2.67 was used to calculate LD as squared allele frequency (*r* ^2^) and physical distance (D) between each pair of SNPs using a sliding window size of 50 with 1,000 permutations. Pairwise LD (*r* ^2^) values were plotted against the relative physical distances (D), and a locally weighted polynomial curve regression (LOESS) model was fitted to determine genome-wide LD decay using the R package (https://www.r-project.org/). The LDs were calculated for the whole wheat genome and three sub-genomes (A, B, D) (Hill and Weir 1988; Remington et al., 2001). The critical value of *r* ^2^ beyond which LD likely starts to decay was set at *r* ^2^ = 0.1. This critical threshold value of *r* ^2^ was estimated using the 95th percentile in the distribution of *r* ^2^ below which the relationship among pairs of marker loci is not caused by physical linkage. The intersection of the fitted curve among correlation *r* ^2^ and physical distance (D) on a chromosome with this critical threshold was considered the estimated LD range. Population kinship heat matrix among all genotypes was calculated using GAPIT in the R package (Wang et al., 2014).

## Results

### Genomic Distribution of Single Nucleotide Polymorphisms

The panel of 184 wheat accessions generated 202,147,814 sequence reads and 129,180 SNPs with 80% missing data points. A total of 123,596 polymorphic SNPs were retained after removing low-quality SNPs with MAF <0.01, heterozygote rate >0.2, and imputation using the Chinese spring wheat reference genome RefSeq V2.0 (IWGSC, 2018) for downstream analysis.

**FIGURE 1:**
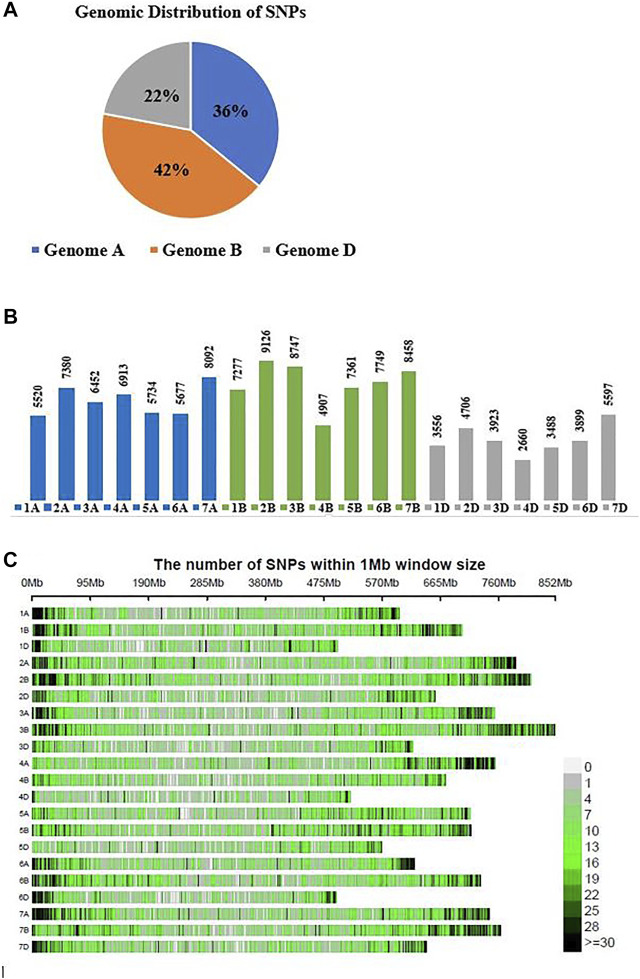
**(A)** Sub-genomic distribution of 123,596 SNPs on three sub-genomes of spring wheat. **(B)** Chromosomal SNPs distribution on each chromosome of three sub-genomes (A, B, D), **(C)** Number of SNPs within 1 Mb window size.

A total of 51,975 SNPs were mapped on the B genome, 44,400 on the A genome, and 27,221 on the D genome (Figure 1A). These results indicated that the B sub-genome of bread wheat holds the highest number of mapped markers (SNPs) followed by the A and D sub-genomes. However, the genome sequence sizes of all sub-genome are nearly equal to 5.5 Gb each. Chromosome 2B has the highest number of mapped SNPs (9,126), while chromosome 4D holds the lowest number of mapped SNPs (2,660) (Figure 1B). The chromosomal distribution of SNPs within 1 Mb window size is presented in Figure 1C, which depicts a higher density of SNPs on chromosomal arms rather than its centromeric region.

### Population Structure

**FIGURE 2:**
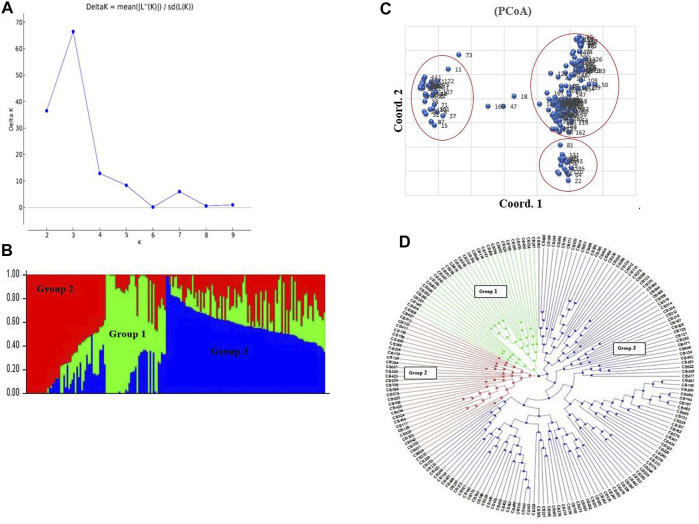
Population structure analysis, **(A)** Delta K line graph, **(B)** structure analysis combine bar chart, **(C)** PCoA analysis of wheat accessions, **(D)** Neighbor Joining phylogenetic tree analysis.

A total of 15,779 highly polymorphic SNPs markers with PIC values ranging from 0.30 to 0.37 were used for downstream data analysis. Population structure analysis was performed on 184 Pakistani wheat accessions, distributed into three sub-groups (group 1, group 2, and group 3) based on optimum K = 3. Delta K value gives the optimal number of subpopulations by plotting a graph with the number of clusters (K) (Figures 2A,B). Results of the STRUCTURE analysis were further confirmed by the PCoA analysis (Figure 2C), which was also supported by neighbor-joining phylogenetic tree analysis (Figure 2D).

**FIGURE 3:**
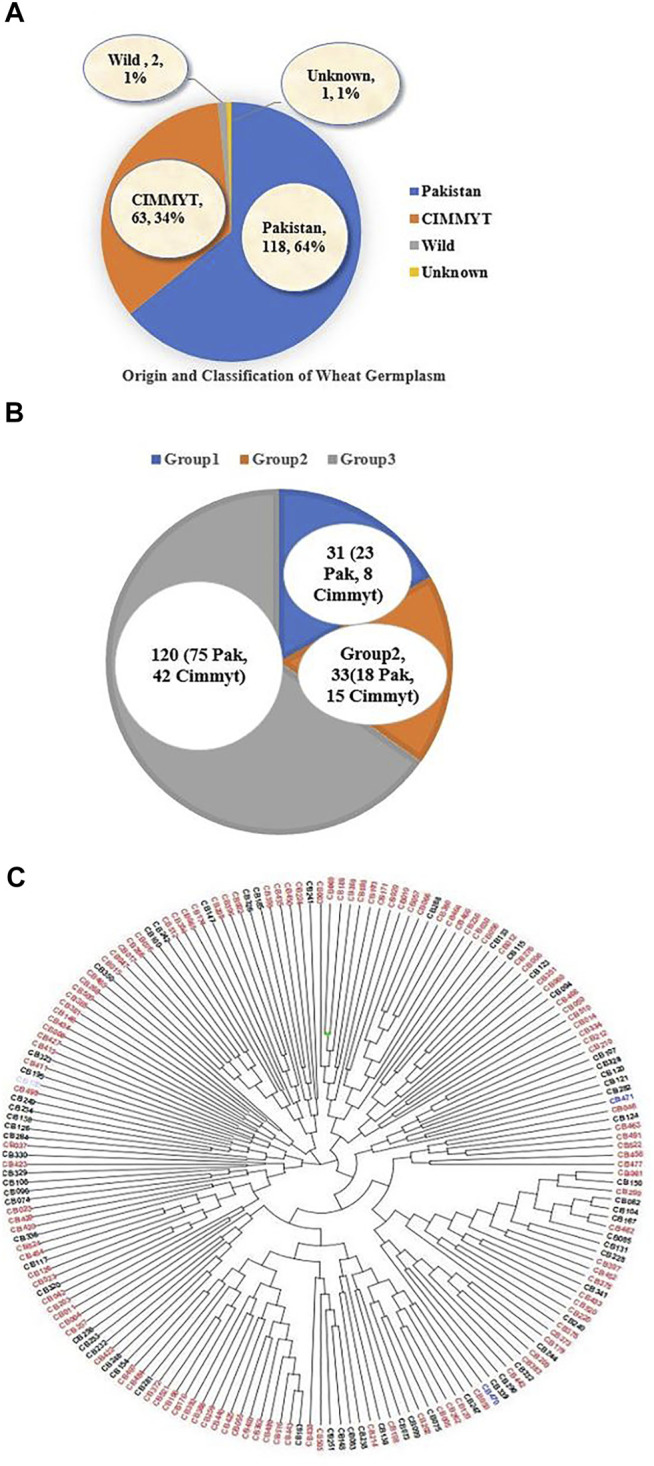
**(A)** Origin of wheat accessions, **(B)** Classification of accessions in three different groups on the basis of structure analysis and phylogenetic tree analysis **(C)** Distribution of different accession in different sub-groups, the red color showed local genotypes, black color indicated CIMMYT accessions, blue color for wild type and purple color for unknown origin.

The panel of 184 accessions was used in this study that includes 118 Pakistani local and 63 accessions from CIMMYT (Centro Internacional de Mejoramiento de Maíz y Trigo; English: International Maize and Wheat Improvement Center, Mexico), two wild wheat relatives, and one accession with unknown origin (Figure 3A). Phylogenetic analysis divided the 184 accessions into three different clusters. Group 1 contained 31 accessions, those dominated by local lines including 23 Pakistani accessions, and only eight CIMMYT accessions. Group 2 consisted of 33 accessions, including 18 Pakistani and 15 from CIMMYT. Group 3 was the largest group that had 120 accessions, including 75 local accessions from Pakistan, 42 from CIMMYT, two wild wheat relatives, and one accession with unknown origin (Figure 3B). A total of 120 local wheat accessions, 63 from CIMMYT, two wild relatives of wheat, and one accession of unknown origin were presented with red, black, blue, and purple colors, respectively (Figure 3C).

### Major Allele Frequency, Genetic Diversity, Heterozygosity, Polymorphic Information Content, and Kinship Heat Map Analysis

Major allele frequency (MF), genetic diversity (GD), heterozygosity (HZ), and the polymorphic information content (PIC) of the wheat panel are listed in Table 1. The whole-genome mean MF was 0.87, with 0.86, 0.87, and 0.89 for A, B, and D sub-genomes, respectively. Mean GD was observed as 0.20, 0.19, and 0.16 for sub-genomes A, B, and D, respectively. The HZ was 0.07, 0.06, and 0.08 for A, B, and D genomes, respectively, and the PIC value was observed as 0.16, 0.16, and 0.14 for A, B, and D sub-genomes, respectively, with 0.15 cumulative PIC for whole wheat genome. Results of the current experiment showed that subgenomes A and B do not have any significant differences among MF, GD, HZ, and PIC values. However, the D sub-genome showed a higher and significant difference among the MF, HZ, relatively lower GD, and PIC values compared to the A and B sub-genomes (Table 1).

**TABLE 1:** Mean major allele frequency, genetic diversity, heterozygosity, and polymorphic information content calculated using 123,596 genome-wide SNPs derived from the panel of 184 wheat genotypes.

Chromosome	Sample size	Marker	MF^a^	GD^b^	HZ^c^	PIC^d^
1A	184	5,344	0.8557	0.2118	0.0763	0.1776
2A	184	7,166	0.8628	0.2055	0.0750	0.1733
3A	184	6,253	0.8720	0.1956	0.0713	0.1660
4A	184	6,668	0.8713	0.1949	0.0731	0.1652
5A	184	5,556	0.8730	0.1938	0.0642	0.1648
6A	184	5,496	0.8696	0.1966	0.0704	0.1667
7A	184	7,889	0.8641	0.2025	0.0679	0.1705
Means			**0.8669**	**0.2001**	**0.0712**	**0.1692**
1B	184	7,062	0.8730	0.1954	0.0651	0.1664
2B	184	8,146	0.8592	0.2114	0.0645	0.1783
3B	184	8,513	0.8710	0.1957	0.0634	0.1660
4B	184	4,700	0.8927	0.1721	0.0630	0.1491
5B	184	7,086	0.8621	0.2067	0.0655	0.1744
6B	184	7,549	0.8628	0.2046	0.0658	0.1723
7B	184	8,218	0.8751	0.1900	0.0607	0.1614
Means			**0.8708**	**0.1965**	**0.0640**	**0.1668**
1D	184	3,466	0.8844	0.1779	0.0902	0.1523
2D	184	4,588	0.8873	0.1779	0.0862	0.1534
3D	184	3,847	0.8995	0.1592	0.0839	0.1377
4D	184	2,591	0.9018	0.1571	0.0868	0.1366
5D	184	3,389	0.8983	0.1623	0.0836	0.1407
6D	184	3,850	0.8869	0.1742	0.0902	0.1492
7D	184	5,490	0.9005	0.1567	0.0798	0.1355
Means			**0.8941**	**0.1665**	**0.0858**	**0.1436**
Genome-wide mean			**0.8772**	**0.1843**	**0.0736**	**0.1598**

**MF** ^**a**^ **:** Major allele frequency, **GD** ^**b**^ **:** Genetic Diversity, **HZ** ^**c**^ **:** Heterozygosity, **PIC** ^**d**^ **:** Polymorphic Information Content.

**FIGURE 4:**
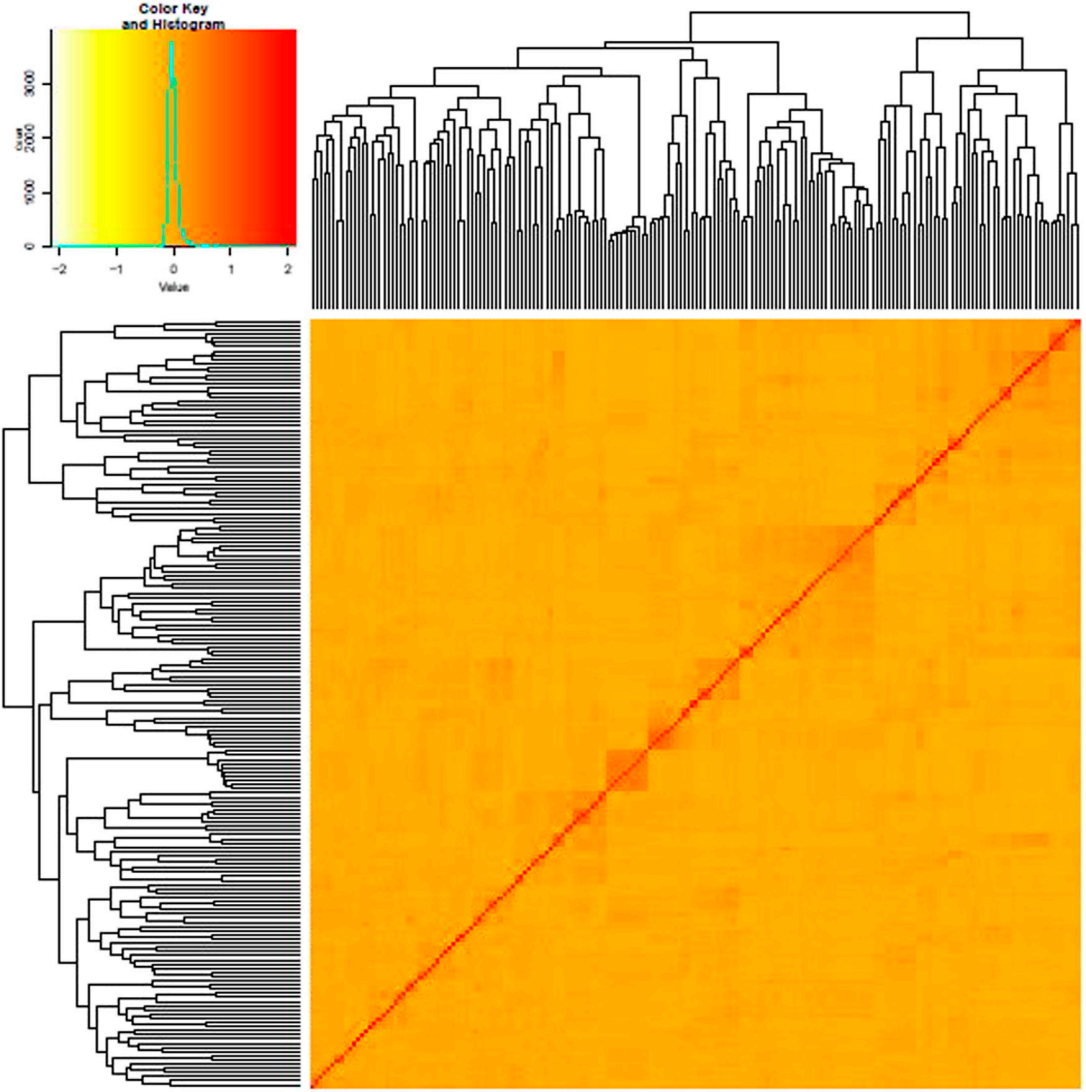
Heat map of kinship matrix with the dendrogram shown on the top-left side based on 123,596 markers.

AMOVA analysis showed 20 and 80% genetic variation among and within the population, respectively. The fixation index F_st_ was used to genetically differentiate total genetic variability among the sub-populations. A low haploid Nm value (0.497) indicates limited gene flow between the sub-populations (Table 2). Shannon’s information index (*I*) also reported very low variation among groups than within different groups (Table 3). The percentage of Shannon’s information index (*I*) and scaled diversity overlapped among and within groups, as presented in Table 3. Kinship analysis also divided the panel into three distinct clusters, suggesting a considerable genetic difference among accessions in this panel. The phylogenetic tree is shown on the top, and the left side of the heat map also confirms the results of structure analysis. However, the intensity of color in the heat map also indicated the high LD regions in the heat map (Figure 4).

**TABLE 2:** Analysis of molecular variance in the panel of 184 genotypes.

Source	df	SS	MS	Est. Var.	%	*p*-value
Among pops	1	16.860	16.860	0.126	20	0.001
Within pops	182	91.502	0.497	0.497	80	0.001
Total	183	108.858	17.357	0.623	100	0.001
Haploid (Nm)	0.49					
Fst	0.67					

Df: degree of freedom, SS: sum of squares, MS: mean sum of squares, %: percentage variation.

Genetic differentiation among and within two subpopulations has been estimated, along with F_st_ gene flow Nm with 9,999 permutations. AP: estimated variance among pops, WP: estimated variance within pops.

***p value <0.001 (based on 9,999 permutations).

**TABLE 3:** Population Shannon information index (*I*).

Source of information	Degrees freedom	Log-like Chi-Sq	Shannon inform	Percent of total	Diversity estimate	[0,1] scaled diversity	[0,1] scaled overlap	Estimated probability
	DF	G-test	*I*	Inform	Exp(*I*)	D′	O’ = 1-D′	*p* (rand ≥ data)
Among pops	1	155.425	0.425	8.604	1.529	0.816	0.184	0.923
Within pops	182	1,651.067	4.511	91.396	91.023	0.998	0.002	0.082
Total	183	1806.492	4.936	100.000	139.180	0.998	0.002	

### Evolution of Linkage Disequilibrium

**FIGURE 5:**
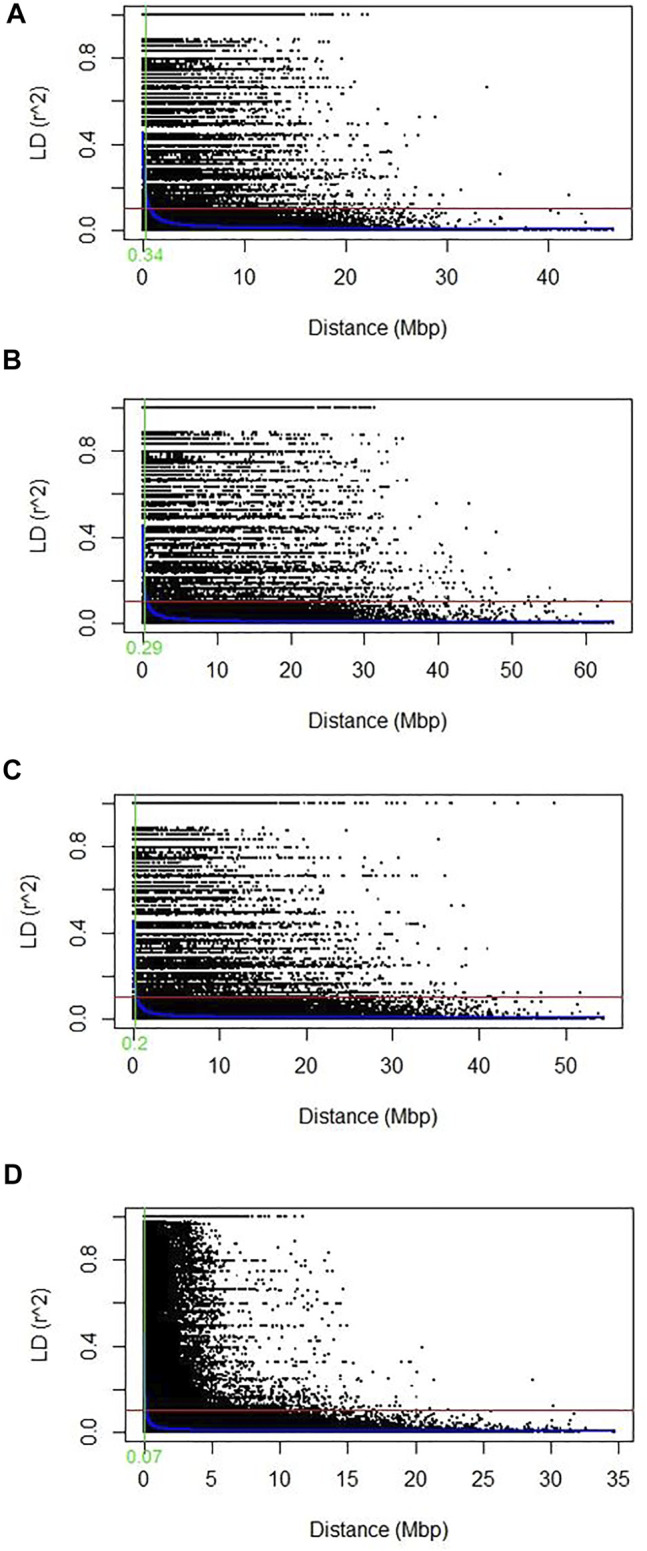
Scatter plot showing the linkage disequilibrium (LD) decay across the genomes for 184 diverse Pakistani bread wheat accessions. The genetic distance in megabase pair (Mbp) plotted against the LD estimate (*r* ^2^) for pairs of SNPs. The solid red line showed the threshold LD value at *r* ^2^ = 0.1 and the solid green line showed the average LD decay point at which the LD curve intercepts the critical *r* ^2^. The LOESS LD decay curve was presented with the solid blue curve **(A)** Genome-wide average LD decay plot using all genomes **(B)** LD decay plot of the A genome **(C)** LD decay plot of the B genome; **(D)** LD decay plot of the D genome.

The scatter plot of (*r* ^2^) and pairwise physical distance (D) revealed that LD decay increases with an increase in marker physical distances on chromosomes or a genome. The average cutoff value *r* ^2^ = 0.1 was used to determine the LD decay level. The LD decay value (0.34 Mbp) was observed for the whole genome (Figure 5A). However, among sub-genomes, the LD decay was the highest on the A subgenome (0.29 Mbp), followed by the B (0.2 Mbp) and D (0.07 Mbp) subgenomes, respectively (Figures 5B–D). Chromosome-wide LD of each sub-genome is shown in Table 4. The LD decay of the A sub-genome ranges from 0.08 to 0.12, and chromosome 3A showed the highest value of LD (0.12). The LD decay of the B sub-genome ranges from 0.05 to 0.13, and chromosome 3B showed a maximum value of LD for the 3B chromosome. However, in the case of the D sub-genome, the LD value ranged from 0.01 to 0.16, and chromosome 2D showed the highest value (0.16) of LD. A low LD value in this population may be due to higher heterozygosity in the sub-genome reflecting the actual hemizygous state of SNPs markers on the D genome.

**TABLE 4:** Chromosome-wise linkage disequilibrium (LD) of individual chromosome.

	A genome	LD (Mbp)	B genome	LD (Mbp)	D genome	LD (Mbp)
1	**A1**	0.09	**B1**	0.05	**D1**	0.12
2	**A2**	0.09	**B2**	0.01	**D2**	0.16
3	**A3**	0.12	**B3**	0.13	**D3**	0.08
4	**A4**	0.08	**B4**	0.08	**D4**	0.01
5	**A5**	0.11	**B5**	0.11	**D5**	0.06
6	**A6**	0.11	**B6**	0.1	**D6**	0.03
7	**A7**	0.1	**B7**	0.11	**D7**	0.06

## Discussion

Genetic diversity among the wheat germplasm is exceptionally important for the genetic improvement of wheat cultivars. Wheat widely grows in South Asia (Pakistan, India, and Bangladesh), producing more than 15% of the world’s wheat production. Pakistan ranks seventh in world wheat production. However, the genetic diversity of most Pakistani wheat germplasm has not been well characterized using genome-wide SNPs markers. Information on the genetic diversity of the germplasm would be crucial for breeding programs that can play a critical role for wheat breeders to make efficient use of available germplasm resources in crop improvement (Hawkes, 1981). In this study, 184 Pakistani spring wheat accessions were genotyped using genome-wide SNPs generated by GBS technology. Most SNPs were mapped on the B genome (42%), followed by the A (36%) and D (22%) sub-genomes (Figure 1A), with the maximum number of SNPs on chromosome 2B (9,126) and the minimum number (2,660) on chromosome 4D (Figure 1B). These findings are consistent with previous studies (Alipour et al., 2017; Bhatta et al., 2017; Eltaher et al., 2018; Rufo et al., 2019; Kumar et al., 2020; Yang et al., 2020).

In the current study, the number of SNPs from the D sub-genome (22%) was about half of these present on the B (42%) sub-genome, which agrees with some of the previous reports that the B genome harbors twice as many of the mapped markers as these present on the D sub-genome (Wang et al., 2013; Iehisa et al., 2014; Edae et al., 2015). However, a much lower ratio of the polymorphic markers was found on the D sub-genome, as low as five times compared to the A or B sub-genomes (Allen et al., 2013; Cavanagh et al., 2013). Low polymorphism in the D genome in most of the germplasm was observed not only for SNPs markers but also for RFLP, AFLP, SSR, and DArT markers (Liu and Tsunewaki, 1991; Röder et al., 1998; Peng et al., 2000; Chao et al., 2007; Akhunov et al., 2010; Würschum et al., 2011; Poland et al., 2012a; Berkman et al., 2013; Marcussen et al., 2014; Nielsen et al., 2014; Shavrukov et al., 2014; Edae et al., 2015; Alipour et al., 2017; Eltaher et al., 2018; Rufo et al., 2019). In wheat, A and B are predecessor sub-genomes that accumulated more genetic recombination, duplication, mutation, and gene flow events than those in the relatively newer D subgenome (Berkman et al., 2013). These results suggest that Pakistani wheat genotypes, like the Iranian germplasm (Alipour et al., 2017), hold more genetic diversity on the D genome than other sources of germplasm. Therefore it is a valuable source for crop improvement against various climate anomalies (Jia et al., 2013). Pakistani wheat accessions as *T. aestivum* L. ssp. Sphaerococcum indigenous from northern Pakistan and northwestern India an early flowering, yellow rustresistant, semi-dwarf plant with a semispherical grain shape (Percival, 1922). This notion points out the ancestral relationship between global populations of *Puccinia striiformis* f. sp. tritici (*Pst*) and the putative origin of *Pst* in the Himalayan, as well as their neighboring plains and foothill regions. The presence of putative alleles, high level of diversity, ability to have the sex-related structure in (*Pst*) races, and clear signature of recombination further support this hypothesis (Ali et al., 2014). Archeological remains and evolutionary studies indicated that hexaploid spring wheat was already grown in the region of India and Pakistan somewhat between 4000 and 2000 BC, which indicates that the region (India and Pakistan) has the longest cultivation and interactions history with *Pst*. This statement also indicates that Pakistani wheat accessions were evolved with comparatively more recombination and ultimately enriched source of genetic diversity (Tengberg, 1999; Habib et al., 2020).

PIC is another important parameter for the selection of markers for breeding programs. Botstein et al. (1980) coined a scale range from 0 to 1.0 to categorize multilocus markers such as SSR according to their PIC value. In the current study, PIC values ranged from 0.004 to 0.37, which is low to moderately high with the mean of 0.158 for the whole genome that was smaller than 0.25 reported by previous literature (Chao et al., 2009; Novoselović et al., 2016; Alipour et al., 2017; El-Esawi et al., 2018; Eltaher et al., 2018; Alemu et al., 2020; Mourad et al., 2020). Among the three sub-genomes, the mean PIC for the D sub-genome (0.14) was slightly lower than that of the A or B sub-genomes (0.16) (Table 1) due to limited hybridization event and gene flow in *Aegilops tauschii* during evolution (Chao et al., 2009; Lopes et al., 2015; Liu Y. et al., 2017; Eltaher et al., 2018; Tyrka et al., 2021). However, much higher PIC (0.26–0.33) were reported in other studies (Allen et al., 2013; Baloch et al., 2017; Chen et al., 2018; Kumar et al., 2020; Yang et al., 2020).

Gene diversity (expected heterozygosity, He) and PIC are considered as primary measures for the dissection of genetic diversity and shedding light on the mutation rate, as well as the evolutionary pressure on a specific allele in a population over the period of time (Botstein et al., 1980; Shete et al., 2000). Overall GD of a population is mainly explained by the distribution of informative markers on a genome (Nielsen et al., 2014). Genetic diversity provides gene diversity of haploid bi-allelic SNPs and ranges of genetic distance, as well as average heterozygosity among individuals of a population (Nei, 1978). As expected, in the current study, the overall mean GD value was greater than PIC (Table 1). In the absence of more polymorphic alleles and ubiquitously even distribution of allele frequency of markers, PIC is always lower than its GD. The PIC values of SNP markers affect the classification of informative markers: highly informative, moderately informative, and slightly informative. Highly informative markers could be used in studying population genetics and GD in various plant species (Salem et al., 2021).

Understanding GD and population structure is a prerequisite to comprehending the genetic variability of germplasm before it can be used in a biotic or abiotic stress breeding program. In the current study, genome-wide mean GD was 0.18, ranging from 0.005 to 0.97 in the panel evaluated, which is slightly lower than 0.26 by Yang et al. (2020) and 0.29 by Mourad et al. (2020) but slightly higher than 0.14 observed by Alipour et al. (2017). The genome-wide heterozygosity (HZ) was 0.0736, ranging from 0 to 0.89. Mean HZ varied greatly among sub-genomes, with the lowest value (0.064) for B and the highest (0.085) for the D sub-genome. A similar trend of HZ values was also reported in other studies (Eltaher et al., 2018; Rimbert et al., 2018; Liu et al., 2019; Kumar et al., 2020).

AMOVA data suggested significant GD within sub-groups (80%), which might result from continuous selection for specific traits under certain environments by wheat breeders. A low level of genetic variability between populations (20%) (Table 2) may be due to schematic gene flow in the form of germplasm exchange among different countries and regions (Arora et al., 2014). The high genetic diversity (80%) within sub-groups means that diversity was attributed to variation within population sub-groups. The results of clustering and AMOVA suggested that inter-cluster cultivars crossing may be useful for developing promising agronomic and disease resistance associated traits in local germplasm. Higher diversity within sub-population than among sub-populations was in line with the findings from other studies (Alipour et al., 2017; Bhatta et al., 2018; Eltaher et al., 2018; Kumar et al., 2020). The Nm value is another important parameter to estimate the gene flow among subpopulations. Nm haploid value of 1 or >1 indicates the low rate of gene flow between subpopulations. In our experiment, the Nm haploid value of 0.497 indicates minor gene flow among three sub-populations because the current germplasm collection does not represent a diverse geographical area. The current population was mainly collected from the Punjab province of Pakistan.

LD is a non-random co-segregation of single/multiple loci among the same or different chromosomes of a genome. The magnitude estimation of LD decay helps determine the resolution of association mapping and the number of SNPs needed for effective association studies and marker-assisted selection (Davey et al., 2011). LD extent varies with sub-genomes or even with chromosomes of the same sub-genome and depends on various factors such as recombination rate, allele frequency, genetic drift, population structure, natural selection, and chromosomal rearrangements (Sukumaran et al., 2015; Habib et al., 2020). Higher values of LD among pairs of markers indicate slower decay rates on a genome or a chromosome, suggesting that fewer markers are needed for GWAS. The current study was conducted on a diverse set of 184 Pakistani spring wheat accessions, and the LD decay at 0.34 Mbp for the whole genome, and 0.29 Mbp, 0.2 Mbp, and 0.07 Mbp for all subgenomes A, B, and D, respectively, observed (Figure 5). In the current study, the A sub-genome showed the highest value of LD (0.29) which shows relatively fewer markers needed for genome coverage and comparatively slower LD decay due to its evolutionary history of origination. However, at the chromosomal level, chromosome 3A showed the highest value (0.12) (Table 4) of LD and chromosome 1A showed the minimum value of LD decay (0.09).

In the case of the B sub-genome chromosomes, 3B and 2B showed maximum and minimum values of LD decay of 0.13 and 0.01, respectively. In the case of D, the sub-genome chromosomes 2D and 4D showed LD values of 0.16 and 0.01, respectively (Table 4). Chromosomal level LD decay helps determine the level of decay, LD hotspots, and number of markers needed for genome coverage on various regions of chromosomes. Results of this study were consistent with the findings of Chao et al. (2010) and Ladejobi et al. (2018) reported the LD decay of 6.4, 4.5, 4, and 4.98 Mbp for A, B, D, and whole-genome, respectively. Aleksandrov et al. (2021) performed a study on Bulgarian bread wheat germplasm to dissect the population structure and linkage disequilibrium on two populations, including one population of old germplasm and one set of modern semi-dwarf cultivars. Old germplasm showed an LD decay of 3.6, 3.3, 3.1, and 3.3 Mb in subgenomes A, B, and D and the whole-genome, respectively. These results support the results of the current study. Li et al. (2019) reported the lowest LD decay value at the D genome, such as 0.35, 0.75, and 0.25 Mb at the A, B, and D subgenomes, respectively. However, the highest LD value was observed in the B sub-genome of the population. Zhang et al. (2013) reported that the highest LD decay rate for the A sub-genome ranges from 25 to 30 cM and on the B and D sub-genomes, LD decay ranges from 15 to 20 cM). Sehgal et al. (2017) reported the LD decay of values of 10, 4, 8, and 5 cM for A, B, D, and whole-genome of the wheat population, respectively. These results also confirm our results of the highest LD decay at the A sub-genome.

Usually, the highest LD value was detected in the D genome in some previous reports (Edae et al., 2015; Sukumaran et al., 2015; Liu W. et al., 2017; Ayana et al., 2018; Bhatta et al., 2018; Jamil et al., 2019; Sandeep et al., 2020). The higher LD value for the D sub-genome might be due to limited and shallow infusion of *Aegilops tauschii* with tetraploid wheat in evolutionary history. The slow LD decay rate in the D sub-genome suggests that more markers may be needed for GWAS, association mapping, and marker-assisted selection (MAS) than those that are needed for the A and B sub-genomes. High LD value in the A sub-genome indicates a few markers needed to cover the genome.

## Conclusion

Analysis of population structure, PIC, LD, PCoA, and NJ phylogenetic tree was used to dissect the genetic diversity of 184 Pakistani genotypes. The whole wheat population was divided into three distinct subpopulations, with group 1 and group 3 dominated by the local lines and group 2 somewhat intermediate type mixture of local and CIMMYT lines. Most of the local genotypes were distributed in all three sub-groups with CIMMYT lines because local accessions were developed by crossing one or both CIMMYT lines as breeding parents. This shows that a huge amount of exotic blood mixing among local germplasm leads toward the ample amount of genetic diversity in local germplasm. Pakistan continuously faces problems such as sudden heatwaves, unusual rainfall, water shortage, and unexpected weather changes due to prompt climate change. Most Pakistani spring wheat genotypes are prone to yellow stripe rust because pathogens get resistant to hot, humid conditions due to unexpected rainfall at the vegetative stage. The results of population structure, phylogenetic tree based genetic distribution, moderately informative nature, and the number of SNP markers, PIC value, and LD regions of this population could be used for the detection of genetic diversity in the local germplasm, and this high genetic diversity would be used for association mapping studies and the selection of crossing parents in national wheat breeding programs. The results of the current genetic diversity study provided the information necessary to broaden the genetic bases and conservation studies. This information will urge national wheat breeders to speed up wheat breeding by using the genetic diversity of local and exotic germplasm. The results of the current study will help dissect dissecting genetic diversity in local germplasm, as well as for GWAS-related studies, to improve the local wheat germplasm of the country.

## Data Availability

The datasets presented in this study can be found in online repositories. The names of the repository/repositories and accession number(s) can be found below: NCBI SRA; PRJNA783303.
