# Supplementary material for: Genotyping-by-Sequencing Based Molecular Genetic Diversity of Pakistani Bread Wheat (Triticum aestivum L.) Accessions
Source: Front Genet. 2022 Apr 6;13:772517. doi: 10.3389/fgene.2022.772517 (PMC9019749; doi:10.3389/fgene.2022.772517)
Supplement: Supplementary file 1 [file Table1.DOCX]

**List of Tables:**

**Table. 1:** Mean major allele frequency, genetic diversity, heterozygosity, and polymorphic information content calculated using 123,596 genome-wide SNPs derived from the panel of 184 wheat genotypes.

| **Chromosome** | **Sample Size** | **Marker** | **MF^a^** | **GD^b^** | **HZ^c^** | **PIC^d^** |
| --- | --- | --- | --- | --- | --- | --- |
| **1A** | 184 | 5,344 | 0.8557 | 0.2118 | 0.0763 | 0.1776 |
| **2A** | 184 | 7,166 | 0.8628 | 0.2055 | 0.0750 | 0.1733 |
| **3A** | 184 | 6,253 | 0.8720 | 0.1956 | 0.0713 | 0.1660 |
| **4A** | 184 | 6,668 | 0.8713 | 0.1949 | 0.0731 | 0.1652 |
| **5A** | 184 | 5,556 | 0.8730 | 0.1938 | 0.0642 | 0.1648 |
| **6A** | 184 | 5,496 | 0.8696 | 0.1966 | 0.0704 | 0.1667 |
| **7A** | 184 | 7,889 | 0.8641 | 0.2025 | 0.0679 | 0.1705 |
| **Means** |  |  | **0.8669** | **0.2001** | **0.0712** | **0.1692** |
| **1B** | 184 | 7,062 | 0.8730 | 0.1954 | 0.0651 | 0.1664 |
| **2B** | 184 | 8,146 | 0.8592 | 0.2114 | 0.0645 | 0.1783 |
| **3B** | 184 | 8,513 | 0.8710 | 0.1957 | 0.0634 | 0.1660 |
| **4B** | 184 | 4,700 | 0.8927 | 0.1721 | 0.0630 | 0.1491 |
| **5B** | 184 | 7,086 | 0.8621 | 0.2067 | 0.0655 | 0.1744 |
| **6B** | 184 | 7,549 | 0.8628 | 0.2046 | 0.0658 | 0.1723 |
| **7B** | 184 | 8,218 | 0.8751 | 0.1900 | 0.0607 | 0.1614 |
| **Means** |  |  | **0.8708** | **0.1965** | **0.0640** | **0.1668** |
| **1D** | 184 | 3,466 | 0.8844 | 0.1779 | 0.0902 | 0.1523 |
| **2D** | 184 | 4,588 | 0.8873 | 0.1779 | 0.0862 | 0.1534 |
| **3D** | 184 | 3,847 | 0.8995 | 0.1592 | 0.0839 | 0.1377 |
| **4D** | 184 | 2,591 | 0.9018 | 0.1571 | 0.0868 | 0.1366 |
| **5D** | 184 | 3,389 | 0.8983 | 0.1623 | 0.0836 | 0.1407 |
| **6D** | 184 | 3,850 | 0.8869 | 0.1742 | 0.0902 | 0.1492 |
| **7D** | 184 | 5,490 | 0.9005 | 0.1567 | 0.0798 | 0.1355 |
| **Means** |  |  | **0.8941** | **0.1665** | **0.0858** | **0.1436** |
| **Genome-wide Mean** |  |  | **0.8772** | **0.1843** | **0.0736** | **0.1598** |

**MF^a^:** Major allele frequency, **GD^b^:** Genetic Diversity, **HZ^c^:** Heterozygosity, **PIC^d^:** Polymorphic Information Content.

**Table. 2** Analysis of molecular variance in the panel of 184 genotypes

| **Source** | **df** | **SS** | **MS** | **Est. Var.** | **%** | **P value** |
| --- | --- | --- | --- | --- | --- | --- |
| **Among Pops** | 1 | 16.860 | 16.860 | 0.126 | 20 | 0.001 |
| **Within pops** | 182 | 91.502 | 0.497 | 0.497 | 80 | 0.001 |
| **Total** | 183 | 108.858 | 17.357 | 0.623 | 100 | 0.001 |
| **Haploid (Nm)** | 0.49 |  |  |  |  |  |
| **F_st_** | 0.67 |  |  |  |  |  |

df: Degree of freedom, SS: Some of square, MS: Mean some of square, %: percentage variation.

Genetic differentiation among and within two subpopulations has been estimated, along with F_st._

gene flow Nm with 9999 permutations

*AP* estimated. variance. among pops, *WP* estimated. variance. within pops

****P* value < 0.001 (based on 9999 permutations)

**Table 3.** Population Shannon information index (*I*)

| **Source of Information** | **Degrees**  **Freedom** | **Log-Like.**  **Chi-Sq** | **Shannon**  **Inform.** | **Percent**  **of Total** | **Diversity**  **Estimate** | **[0,1] Scaled**  **Diversity** | **[0,1] Scaled**  **Overlap** | **Estimated**  **Probability** |
| --- | --- | --- | --- | --- | --- | --- | --- | --- |
|  | **DF** | **G-Test** | ***I*** | **Inform.** | **Exp(*I*)** | **D'** | **O'=1-D'** | **P(rand >= data)** |
| **Among Pops** | 1 | 155.425 | 0.425 | 8.604 | 1.529 | 0.816 | 0.184 | 0.923 |
| **Within Pops** | 182 | 1651.067 | 4.511 | 91.396 | 91.023 | 0.998 | 0.002 | 0.082 |
| **Total** | 183 | 1806.492 | 4.936 | 100.000 | 139.180 | 0.998 | 0.002 |  |

**Table. 4:** Chromosome wise linkage Disequilibrium (LD) of individual chromosome.

|  | **A genome** | **LD (Mbp)** | **B genome** | **LD (Mbp)** | **D genome** | **LD (Mbp)** |
| --- | --- | --- | --- | --- | --- | --- |
| **1** | **1A** | 0.09 | **1B** | 0.05 | **1D** | 0.12 |
| **2** | **2A** | 0.09 | **2B** | 0.01 | **2D** | 0.16 |
| **3** | **3A** | 0.12 | **3B** | 0.13 | **3D** | 0.08 |
| **4** | **4A** | 0.08 | **4B** | 0.08 | **4D** | 0.01 |
| **5** | **5A** | 0.11 | **5B** | 0.11 | **5D** | 0.06 |
| **6** | **6A** | 0.11 | **6B** | 0.1 | **6D** | 0.03 |
| **7** | **7A** | 0.1 | **7B** | 0.11 | **7D** | 0.06 |

**Supplementary Material.**

**Supplementary Table S1.** Pedigree, CB. No, phylogenetic group, origin of Pakistani wheat accessions.

| **Sr. No** | **Pedigree** | **CB. No** | **Phylogenetic**  **Group No** | **Origin** |
| --- | --- | --- | --- | --- |
| 1 | BHAKKAR-2000 | CB3 | 3 | Pakistan |
| 2 | CHAKWAL-50 | CB4 | 2 | Pakistan |
| 3 | FAREED-06 | CB8 | 1 | Pakistan |
| 4 | INQILAB 91 | CB11 | 2 | Pakistan |
| 5 | MANTHAR | CB14 | 3 | Pakistan |
| 6 | MIRAJ-08 | CB15 | 1 | Pakistan |
| 7 | SHAFAQ-06 | CB17 | 1 | Pakistan |
| 8 | BARS-09 | CB20 | 3 | Pakistan |
| 9 | BHITTAI | CB23 | 2 | Pakistan |
| 10 | SASSI | CB30 | 3 | Pakistan |
| 11 | PIRSABAK 2005 | CB37 | 2 | Pakistan |
| 12 | CHENAB-79 | CB42 | 2 | Pakistan |
| 13 | IQBAL2000 | CB46 | 3 | Pakistan |
| 14 | JAUHAR-78 | CB47 | 1 | Pakistan |
| 15 | KOHISTAN 97 | CB50 | 3 | Pakistan |
| 16 | PASINA 90 | CB56 | 3 | Pakistan |
| 17 | PUNJAB-76 | CB57 | 3 | Pakistan |
| 18 | PUNJAB 96 | CB60 | 3 | Pakistan |
| 19 | SA-42 | CB61 | 3 | Pakistan |
| 20 | SHAHKAR 95 | CB65 | 1 | Pakistan |
| 21 | SHALIMAR-88 | CB66 | 3 | Pakistan |
| 22 | ZA-77 | CB69 | 3 | Pakistan |
| 23 | KAUZ'S' | CB73 | 3 | Australia/CIMMYT |
| 24 | NACOZARI F-76 | CB74 | 2 | CIMMYT |
| 25 | OASIS F-86 | CB75 | 3 | CIMMYT |
| 26 | PBW-343=ATTILA | CB76 | 1 | India/CIMMYT |
| 27 | FRET-1 | CB80 | 3 | CIMMYT |
| 28 | WH-542 | CB82 | 3 | India/CIMMYT |
| 29 | HOOSAM-3 | CB83 | 3 | ICARDA |
| 30 | SAAR | CB85 | 3 | CIMMYT |
| 31 | CHAM-4 | CB94 | 3 | ICARDA |
| 32 | CHILERO=CHIL'S' | CB96 | 2 | CIMMYT |
| 33 | FRONTANA | CB99 | 3 | Brazil/CIMMYT |
| 34 | HARTOG=HTG.(PAVON) | CB100 | 1 | CIMMYT |
| 35 | OASIS/SKAUZ//4*BCN/3/2*PASTOR | CB103 | 3 | CIMMYT |
| 36 | BABAX/LR42//BABAX*2/3/VIVITSI | CB104 | 3 | CIMMYT |
| 37 | PBW 343*2/KUKUNA | CB106 | 2 | CIMMYT |
| 38 | PBW 343*2/KURUKU | CB107 | 3 | CIMMYT |
| 39 | PVN//CAR422/ANA/3/KAUZ*2/TRAP//KAUZ | CB115 | 3 | CIMMYT |
| 40 | TRAP#1/PBW65/3/KAUZ*2/TRAP//KAUZ | CB117 | 2 | CIMMYT |
| 41 | PVN/PBW65/3/KAUZ*2/TRAP//KAUZ | CB120 | 3 | CIMMYT |
| 42 | PARULA=PRL | CB121 | 3 | CIMMYT |
| 43 | NING-8319 | CB123 | 3 | China/CIMMYT |
| 44 | HARRIER 17.B | CB124 | 3 | Australia/CIMMYT |
| 45 | V-03007 | CB126 | 2 | Pakistan |
| 46 | CON.'S'/ANA 75//CON.'S' | CB128 | 2 | CIMMYT |
| 47 | HD2236//SA.42/HARRIER'S= V-97088 | CB129 | 3 | Pakistan |
| 48 | PB81//F3.71/TRM/3/BULBUL// F3. 71/ TRM  =V0005 | CB130 | 3 | CIMMYT |
| 49 | WEEBILL-1 = V-03158 | CB131 | 3 | CIMMYT |
| 50 | WATAN/2*ERA | CB133 | 3 | CIMMYT |
| 51 | TURACO/PRINIA | CB138 | 2 | CIMMYT |
| 52 | PB-96/87094//MH-97 | CB146 | 1 | Pakistan |
| 53 | MAYA/PVN | CB147 | 1 | CIMMYT |
| 54 | WL 711/CROW “S”//ALD #1 / CMH77A.917/3/HI 666/PVN ‘S’ | CB148 | 3 | CIMMYT |
| 55 | PRL/2*PASTOR//PARUS/5/NAC/TH.AC//3*  PVN/3/MIRLO/BUC/4/2*PASTOR | CB150 | 3 | CIMMYT |
| 56 | FRET2*2/4/SNI/TRAP#1/3/KAUZ*2/TRAP//KAUZ/5/ONIX | CB154 | 3 | CIMMYT |
| 57 | CROC-1/AE.SQ(224)//OPATA/3/FLAG-7 | CB163 | 3 | CIMMYT |
| 58 | SERI.1B*2/3/KAUZ*2/BOW//K | CB165 | 1 | CIMMYT |
| 59 | SHUHA-4//NS732/HER/3/ MILAN/DUCULA | CB167 | 3 | CIMMYT |
| 60 | BWP 122526 | CB171 | 3 | Pakistan |
| 61 | NW S-2001 | CB174 | 1 | Pakistan |
| 62 | PR-111 | CB176 | 3 | Pakistan |
| 63 | PR-106 | CB179 | 3 | Pakistan |
| 64 | 13248 | CB186 | 3 | Pakistan |
| 65 | LONG GRAIN | CB188 | 2 | Unknown |
| 66 | NSW-14 | CB195 | 2 | Australia/CIMMYT |
| 67 | V-12266 | CB196 | 3 | Pakistan |
| 68 | V-13270 | CB198 | 2 | Pakistan |
| 69 | 122557 | CB200 | 3 | Pakistan |
| 70 | V-02192 | CB202 | 3 | Pakistan |
| 71 | V-02156 | CB203 | 2 | Pakistan |
| 72 | V-04048 | CB208 | 1 | Pakistan |
| 73 | V-05115 | CB210 | 3 | Pakistan |
| 74 | V-06129 | CB212 | 3 | Pakistan |
| 75 | V-056132 | CB214 | 3 | Pakistan |
| 76 | V-06018 | CB220 | 3 | Pakistan |
| 77 | V-06068 | CB224 | 1 | Pakistan |
| 78 | KIRITATI//PBW65/2*SERI.1B | CB228 | 3 | CIMMYT |
| 79 | KIRITATI/4/2*SERI.1B*2/3/KAUZ*2/BOW//KAUZ | CB232 | 3 | CIMMYT |
| 80 | WHEAR/VIVITSI//WHEAR | CB234 | 2 | CIMMYT |
| 81 | WHEAR/CHAPIO//WHEAR | CB236 | 3 | CIMMYT |
| 82 | WHEAR/KUKUNA/3/C80.1/3*BATAVIA//2*WBLL1 | CB238 | 3 | CIMMYT |
| 83 | INQALAB91*2/KUKUNA// | CB239 | 3 | CIMMYT |
| 84 | SUNCO//TNMU/TUI | CB240 | 1 | CIMMYT |
| 85 | SHARP/3/PRL/SARA//TSI/VEE#5/5/VEE/LIRA//BOW/3/  BCN/4/KAUZ | CB242 | 1 | CIMMYT |
| 86 | DOLLARBIRD | CB244 | 3 | Australia/CIMMYT |
| 87 | KIRITATI | CB247 | 3 | CIMMYT |
| 88 | PFAU/WEAVER*2//KIRITATI | CB248 | 3 | CIMMYT |
| 89 | PGO/SERI//BAV92 | CB249 | 2 | CIMMYT |
| 90 | KINGBIRD#1 | CB251 | 3 | CIMMYT |
| 91 | TAM200/TUI | CB253 | 3 | CIMMYT |
| 92 | CROC_1/AE.SQUARROSA (205)//  FCT/3/PASTOR | CB256 | 3 | CIMMYT |
| 93 | HD 2169/C591//PBW343 | CB259 | 3 | Pakistan |
| 94 | V-86711TC/SH-88//CROW | CB262 | 3 | Pakistan |
| 95 | AS2002/WL711//SHAFAQ | CB266 | 1 | Pakistan |
| 96 | INQ91/YR-31 | CB268 | 1 | Pakistan |
| 97 | INQ91/YR-31 | CB269 | 3 | Pakistan |
| 98 | V-04179/T7 (T.SPHAEROCOCCUM) –DROUGHT | CB276 | 3 | Pakistan |
| 99 | WBLLI*2/VIVITSI/3/T.DICOCCOMP194624  /AE.SQ(409)//BCN/4/WBLL1*2/VIVTSI/5/WBLLI | CB281 | 3 | CIMMYT |
| 100 | WBLLI*2/VIVITSI/3/T.DICOCCOMP194624/  AE.SQ(409)//BCN/4/WBLL1*2/VIVTSI/5/WBLLI | CB282 | 3 | CIMMYT |
| 101 | KAUZ//ALTAR84/AOS/3/MILAN/KAUZ/4/HUTES  /5/T.SPELTAP1384764/6/2*KAUZ//ALTAR84/AOS | CB284 | 2 | CIMMYT |
| 102 | TOBA97/PASTOR*2//T.SPELTA P1348774 | CB288 | 3 | CIMMYT |
| 103 | T.SPELTA P1348764//INQ.91*2/TUKORU/3/WBLL1*2/TUKURU | CB290 | 3 | CIMMYT |
| 104 | V-11186 | CB320 | 2 | CIMMYT |
| 105 | MUNAL #1 | CB323 | 2 | CIMMYT |
| 106 | TACUPETO F2001/BRAMBLING//KIRITATI | CB326 | 1 | CIMMYT |
| 107 | ATTILA/3*BCN//BAV92/3/TILHI/5/BAV92/3/PRL/SARA  //TSI/VEE#5/4/CROC_1/AE.SQUARROSA (224)//2*OPATA | CB328 | 3 | CIMMYT |
| 108 | ROLF07*2/KIRITATI | CB329 | 2 | CIMMYT |
| 109 | FRET2/KUKUNA//FRET2/3/PARUS/5/FRET2*2/4  /SNI/TRAP#1/3/KAUZ*2/TRAP//KAUZ | CB330 | 2 | CIMMYT |
| 110 | PBW343*2/KUKUNA*2//YANAC | CB332 | 3 | CIMMYT |
| 111 | TRCH//PRINIA/PASTOR | CB334 | 3 | CIMMYT |
| 112 | ACHTAR*3//KANZ/KS85-85/4/MILAN/KAUZ//PRINIA  /3/BAV92/5/MILAN/KAUZ//PRINIA/3/BAV92 | CB336 | 2 | CIMMYT |
| 113 | SOKOLL*2/TROST | CB339 | 3 | CIMMYT |
| 114 | VILLA JUAREZ F2009/SOLALA//WBLL1*2/BRAMBLING | CB341 | 3 | CIMMYT |
| 115 | NR 388 | CB350 | 1 | CIMMYT |
| 116 | NR-371 | CB351 | 3 | Pakistan |
| 117 | NR-379 | CB354 | 1 | Pakistan |
| 118 | NR-390 | CB357 | 3 | Pakistan |
| 119 | NR-403 | CB362 | 3 | Pakistan |
| 120 | 76377 | CB369 | 3 | Pakistan |
| 121 | 99108 | CB372 | 3 | Pakistan |
| 122 | V-11183 –RF | CB373 | 3 | Pakistan |
| 123 | V-11365 | CB375 | 3 | Pakistan |
| 124 | 11B2049 | CB378 | 3 | Pakistan |
| 125 | 11BT004 | CB381 | 1 | Pakistan |
| 126 | V-11143 | CB383 | 3 | Pakistan |
| 127 | V-12284 | CB385 | 1 | Pakistan |
| 128 | NW-10-1111-7 | CB387 | 3 | Pakistan |
| 129 | V-11046 | CB388 | 3 | Pakistan |
| 130 | NS-10 | CB390 | 3 | Pakistan |
| 131 | NR 411 | CB392 | 3 | Pakistan |
| 132 | V-11001 | CB396 | 1 | Pakistan |
| 133 | 12257 | CB399 | 1 | Pakistan |
| 134 | 12292 | CB400 | 3 | Pakistan |
| 135 | D67.2/PARANA 66.270//AE.SQ (320)/3/CUNNINGHAM/4/  VORB | CB403 | 3 | Pakistan |
| 136 | ATTILA*2/PBW65*2//HAWFINCH #1 | CB411 | 2 | Pakistan |
| 137 | PFAU/SERI.1B//AMAD/3/WAXWING*2/4/MUU | CB413 | 2 | Pakistan |
| 138 | V-12253 | CB420 | 2 | Pakistan |
| 139 | V-11160 | CB422 | 3 | Pakistan |
| 140 | 12266 | CB423 | 2 | Pakistan |
| 141 | 11138 | CB425 | 3 | Pakistan |
| 142 | V-13005 | CB427 | 2 | Pakistan |
| 143 | V-13016 | CB429 | 2 | Pakistan |
| 144 | V-12130 | CB433 | 3 | Pakistan |
| 145 | V-12057 | CB434 | 1 | Pakistan |
| 146 | V-13241 | CB435 | 1 | Pakistan |
| 147 | V-13255 | CB438 | 3 | Pakistan |
| 148 | V-12066 | CB440 | 3 | Pakistan |
| 149 | V-13266 | CB442 | 3 | Pakistan |
| 150 | V-13270 | CB443 | 3 | Pakistan |
| 151 | 122557 | CB450 | 1 | Pakistan |
| 152 | 112095 | CB452 | 3 | Pakistan |
| 153 | 12BT012 | CB454 | 2 | Pakistan |
| 154 | 12C027 | CB456 | 3 | Pakistan |
| 155 | TW /424 | CB462 | 3 | Pakistan |
| 156 | TWS12268 | CB463 | 3 | Pakistan |
| 157 | MSW | CB464 | 3 | Pakistan |
| 158 | NR-429 | CB465 | 1 | Pakistan |
| 159 | NR-449 | CB466 | 3 | Pakistan |
| 160 | TRITICUMPYRUM (V-2) | CB470 | 3 | Wild relative |
| 161 | TRITICUMPYRUM (V-3) | CB471 | 3 | Wild relative |
| 162 | F6 3013 (BWP) | CB477 | 3 | Pakistan |
| 163 | 088200 (MONO TILLER EARLY MATURITY WITH LESS LODGING) | CB484 | 3 | Pakistan |
| 164 | YOUNIS | CB488 | 3 | Pakistan |
| 165 | 13B-3146 | CB491 | 3 | Pakistan |
| 166 | TWS-12464 | CB493 | 3 | Pakistan |
| 167 | NR-443 | CB497 | 3 | Pakistan |
| 168 | NR-487 | CB500 | 1 | Pakistan |
| 169 | 14C036 | CB505 | 3 | Pakistan |
| 170 | NIBGE GANDUM N | CB508 | 3 | Pakistan |
| 171 | 13BT016 | CB512 | 1 | Pakistan |
| 172 | 14152 | CB516 | 3 | Pakistan |
| 173 | 14170 | CB520 | 3 | Pakistan |
| 174 | 14168 | CB521 | 3 | Pakistan |
| 175 | 13167 | CB522 | 3 | Pakistan |
| 176 | 14227 | CB523 | 2 | Pakistan |
| 177 | 13338 | CB524 | 2 | Pakistan |
| 178 | AUQAB-2000 | CB2 | 1 | Pakistan |
| 179 | CHAKWAL-97 | CB6 | 3 | Pakistan |
| 180 | UFAQ | CB19 | 3 | Pakistan |
| 181 | CHAKWAL-86 | CB5 | 3 | Pakistan |
| 182 | PUNJAB-85 | CB59 | 3 | Pakistan |
| 183 | KOHISAR-95 | CB12 | 3 | Pakistan |
| 184 | PARVAZ-94 | CB55 | 3 | Pakistan |
